# Methotrexate myelopathy after intrathecal chemotherapy: a case report

**DOI:** 10.1186/s13256-015-0597-5

**Published:** 2015-06-09

**Authors:** Ken-ya Murata, Ayaka Maeba, Mika Yamanegi, Ichiro Nakanishi, Hidefumi Ito

**Affiliations:** Department of Neurology, Wakayama Medical University, 840-1 Kimii-dera, Wakayama, Wakayama 641-8510 Japan

**Keywords:** Intrathecal chemotherapy, Malignant lymphoma, Methotrexate myelopathy, S-adenosylmethionine, Subacute combined degeneration

## Abstract

**Introduction:**

Methotrexate is often administered intrathecally or into the cerebral ventricles, particularly in patients with central nervous system tumors. However, in addition to chemical arachnoiditis, methotrexate can induce severe myelopathy.

**Case presentation:**

A 59-year-old Japanese man with diffuse B-cell lymphoma who underwent systemic chemotherapy including methotrexate and 20Gy of radiotherapy received intrathecal methotrexate for recurrence. Flaccid paresis of his lower limbs and fecal and urinary incontinence appeared 1 month later. All sensations were impaired below the Th10 dermatome level. Although the clinical symptoms were compatible with transverse myelitis, T2-weighted imaging of his thoracic spinal cord demonstrated signal hyperintensity localized to the posterior and lateral funiculi, which resembled subacute combined degeneration. His serum vitamin B12, folic acid, and total homocysteine levels were within normal limits, but total homocysteine levels in his cerebrospinal fluid were elevated, suggesting spinal cord demyelination.

**Conclusions:**

Little is known of the pathogenesis of methotrexate myelopathy. A possible mechanism of methotrexate myelopathy with demyelination was suggested by the increased homocysteine levels in the cerebrospinal fluid.

## Introduction

Methotrexate (MTX) suppresses DNA synthesis and proliferation of tumor cells by preventing the conversion of dihydrofolate to tetrahydrofolate (THF) [1]. MTX shows minimal transfer through the blood–brain barrier of the central nervous system (CNS), thus, it is often administered intrathecally. MTX can induce confusion, headaches, or seizures in the acute phase and severe myelopathy in the subacute phase [1]. Here, we report the case of a patient who developed transverse myelitis after intrathecal MTX therapy for malignant lymphoma and postulate a mechanism for MTX myelopathy.

## Case presentation

A 59-year-old Japanese man complained of pain in his lower right leg. Abdominal magnetic resonance imaging (MRI) revealed a right pelvic tumor compatible with histological findings of non-Hodgkin diffuse B-cell lymphoma. CODOX-M/IVAC (CODOX-M: cyclophosphamide, vincristine, doxorubicin, and high-dose MTX; IVAC: ifosfamide, etoposide, and high-dose cytarabine) was started, and shrinkage of the tumor was achieved. At 5 months after CODOX-M/IVAC therapy, he complained of dysesthesia of his bilateral feet. He began taking methylcobalamin, but there was no improvement. Spinal MRI revealed another mass anterior to the 2nd to 3rd sacral vertebral bodies. Cerebrospinal fluid (CSF) cytology showed malignant findings after 20Gy radiation therapy. CODOX-M/IVAC was administered a second time, and two administrations of intrathecal MTX (15mg) were started, with the addition of calcium folinate. Because flaccid paresis of his lower limbs and fecal and urinary incontinence appeared 1 month later, he was referred to our department.

His blood pressure was 102/72mmHg and body temperature was 36.7°C. A neurological examination revealed that he was alert and well oriented. His mental status was normal, and his cranial nerves appeared intact. He showed flaccid paresis of his lower limbs with an absence of tendon reflexes and both fecal and urinary incontinence. Extensor plantar responses were noted on the left side. Pinprick, light touch, vibration, and proprioception sensations were impaired below the Th10 dermatome level. Cerebellar ataxia was not observed.

Laboratory results revealed normocytic anemia. His serum vitamin B12 levels were 2788pg/mL and all other tests showed unremarkable results (copper, 132μg/dL; total homocysteine, 8.7nmol/mL; and folic acid, 7.6ng/dL). A CSF examination showed cytoalbuminic dissociation (mononuclear cells, 1/mm^3^; protein, 123mg/dL) and a glucose level of 47mg/dL (blood sugar, 98mg/dL). Myelin basic protein levels were 3087pg/mL and his CSF homocysteine levels were 1.2nmol/mL. Negative results were obtained for CSF oligoclonal bands, soluble interleukin-2 receptor, CSF cytology, and polymerase chain reaction testing for various viruses. Our patient denied all genetic analysis including homocysteine metabolism.

**Fig. 1:**
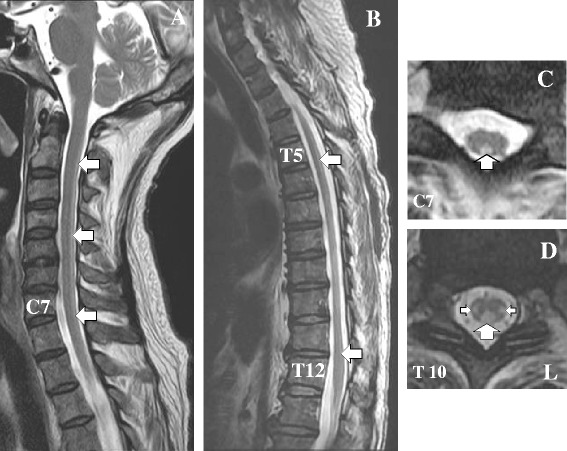
Conventional T2-weighted magnetic resonance imaging of the cervical and thoracic spinal cord after 6 weeks of intrathecal administration of methotrexate. **a** Sagittal image shows signal hyperintensity (arrows) in the cervical spine from C2 to C7. **b** Sagittal image shows signal hyperintensity (arrows) in the thoracic spine from Th4 to Th11. **c** Coronal image shows signal hyperintensity in the posterior column at the C7 level (arrow). **d** Coronal image shows signal hyperintensity in bilateral dorsal columns at the Th10 level (arrows)

Findings from brain MRI were unremarkable, but spinal MRI revealed signal hyperintensity on T2-weighted imaging in the posterior funiculus of his cervical spinal cord and in the lateral and posterior funiculi of his thoracic spinal cord (Fig. 1).

Leucovorin calcium at 60mg/day and high-dose vitamin B12 replacement therapy were administered for 2 weeks, but no improvements were observed. He died 3 months later due to progression of the primary disease.

## Discussion

In this study, we described a 59-year-old man diagnosed with non-Hodgkin diffuse B-cell lymphoma. He showed symptoms of transverse myelitis after intrathecal MTX therapy. MRI findings in the dorsal and lateral columns along with the caudal-to-rostral extension of affected regions resembled those of subacute combined degeneration (SCD). The clinical features of SCD are pyramidal signs and impaired deep sensation. Our patient showed transverse myelitis, which is very rare in patients with SCD. Thus, his clinical and MRI findings indicated MTX myelopathy (Table 1).

**Table 1:** **Methotrexate myelopathy exhibiting subacute combined degeneration-like image findings**

**Patient number**	**Age (years)/Sex**	**Diagnosis**	**Total dose of intrathecal methotrexate**	**Methotrexate therapy before intrathecal administration**	**Clinical course**	**Symptoms of transverse myelitis**	**Signal hyperintensity on T2-weighted magnetic resonance imaging**	**Serum vitamin B12/folic acid**	**Clinical improvement after vitamin supplementation**	**Reference**
1	28/F	NHL	12mg ×8	+	Subacute	+	C-S (posterior and lateral funiculi)	Normal/normal	Mild	Lu *et al*. [8]
2	60/F	NHL	12mg ×9	–	Subacute	–	C-S (posterior funiculus)	Normal/N.D.	No	Lu *et al*. [8]
3	26/F	ALL	12mg ×3	+	Acute	+	C-T (posterior funiculus)	Normal/N.D.	Mild	Counsel and Khangure [9]
4	45/M	ALL	15mg ×7	+	Subacute	+	T-S (posterior and lateral funiculi)	N.D.	No	Honda and Ujihira [10]
5	42/F	ALL	12mg ×2	–	Subacute	–	C-T (posterior and lateral funiculi)	High/normal	No	Gosavi *et al*. [11]
6	59/M	NHL	14mg ×5	+	Subacute	+	C-T (posterior and lateral funiculi)	Normal/normal	No	Our study

ALL, acute lymphocytic leukemia; C, cervical spinal cord; F, female; M, male; N.D., not determined; NHL, non-Hodgkin lymphoma; S, sacral spinal cord; T, thoracic spinal cord

Vacuolar degeneration in the white matter without inflammatory cell infiltration is the main pathological finding of MTX myelopathy, according to postmortem studies. Indeed, demyelination is most prominent in the posterior funiculus, but has been observed in both the lateral and anterior funiculi [2]. Furthermore, demyelination is severe at the surface of the spinal cord in contact with the CSF, whereas white matter lesions in the center of the spinal cord appear mild [3]. Such findings suggest that the demyelination starts from the surface of the spinal cord and progresses centrally. Even MRI findings appear similar to SCD, and pathological findings show demyelination of the entire cross-section of the spinal cord, resulting in transverse myelopathy.

Intrathecal MTX administration inhibits the production of THF and decreases intrathecal 5-methyl-THF production [4]. The methyl residue from 5-methyl-THF is required to convert cobalamin into methylcobalamin, and methylcobalamin is essential for the synthesis of methionine from homocysteine. Homocysteine levels in CSF are normally ≤0.5nmol/mL in healthy individuals [5], and can increase up to 1.0nmol/mL with systemic MTX administration [4]. In the present case, the total homocysteine level in CSF increased to 1.2nmol/mL, whereas that in serum remained within normal limits. The increased homocysteine levels in CSF reflect methionine synthesis disruption in the CNS. S-adenosylmethionine (SAM) is synthesized from methionine and is indispensable for the maintenance of the myelin sheath [6]. Intrathecal MTX administration eventually decreases SAM synthesis, and SAM deficiency in turn induces demyelination in the spinal cord [6]. An increased level of myelin basic protein also suggests spinal cord demyelination. Substitution with multiple metabolites may be a promising strategy for the treatment of MTX-induced neurotoxicity; however, we had no chance to try these therapies before the patient died [7].

## Conclusions

We have postulated a possible mechanism of MTX myelopathy with demyelination from the biochemical perspective. However, further studies examining a large number of patients with MTX myelopathy are needed to confirm our hypothesis.

## Consent

Written informed consent was obtained from the patient for publication of this case report and accompanying images. A copy of the written consent is available for review by the Editor-in-Chief of this journal.

## References

[1] Vezmar S, Becker A, Bode U, Jaehde U. Biochemical and clinical aspects of methotrexate neurotoxicity. Chemotherapy. 2003;49:92–104. 10.1159/000069773 12714818

[2] Clark AW, Cohen SR, Nissenblatt MJ, Wilson SK. Paraplegia following intrathecal chemotherapy: neuropathologic findings and elevation of myelin basic protein. Cancer. 1982;50:42–7. 10.1002/1097-0142(19820701)50:1<42::aid-cncr2820500109>3.0.co;2-m 6177392

[3] Bates SE, Raphaelson MI, Price RA, McKeever P, Cohen S, Poplack DG. Ascending myelopathy after chemotherapy for central nervous system acute lymphoblastic leukemia: correlation with cerebrospinal fluid myelin basic protein. Med Pediatr Oncol. 1985;13:4–8. 10.1002/mpo.2950130103 2578602

[4] Becker A, Vezmar S, Linnebank M, Pels H, Bode U, Schlegel U, et al. Marked elevation in homocysteine and homocysteine sulfinic acid in the cerebrospinal fluid of lymphoma patients receiving intensive treatment with methotrexate. Int J Clin Pharmacol Ther. 2007;45:504–15. 10.5414/cpp45504 17907593

[5] Serot JM, Barbe F, Arning E, Bottiglieri T, Franck P, Montagne P, et al. Homocysteine and methylmalonic acid concentrations in cerebrospinal fluid: relation with age and Alzheimer’s disease. J Neurol Neurosurg Psychiatry. 2005;76:1585–7. 10.1136/jnnp.2004.056119 PMC1739416 16227558

[6] Hyland K, Smith I, Bottiglieri T, Perry J, Wendel U, Clayton PT, et al. Demyelination and decreased S-adenosylmethionine in 5,10-methylenetetrahydrofolate reductase deficiency. Neurology. 1988;38:459–62. 10.1212/wnl.38.3.459 3347350

[7] Ackermann R, Semmler A, Maurer GD, Hattingen E, Fornoff F, Steinbach JP, et al. Methotrexate-induced myelopathy responsive to substitution of multiple folate metabolites. J Neurooncol. 2010;97:425–7. 10.1007/s11060-009-0028-9 19821069

[8] Lu CH, Yao M, Liu HM, Chen YF. MR findings of intrathecal chemotherapy-related myelopathy in two cases: mimicker of subacute combined degeneration. J Neuroimaging. 2007;17:184–7. 10.1111/j.1552-6569.2007.00094.x 17441843

[9] Counsel P, Khangure M. Myelopathy due to intrathecal chemotherapy: magnetic resonance imaging findings. Clin Radiol. 2007;62:172–6. 10.1016/j.crad.2006.09.005 17207701

[10] Honda DMS, Ujihira N. An autopsy case of methotrexate (MTX)-induced myelopathy mimicking subacute combined degeneration (SCD). Neuropathology. 2011;31:357.

[11] Gosavi T, Diong CP, Lim SH. Methotrexate-induced myelopathy mimicking subacute combined degeneration of the spinal cord. J Clin Neurosci. 2013;20:1025–6. 10.1016/j.jocn.2012.06.018 23647709

